# An Incidental Finding of Mucinous Colon Cancer by ^18^F-Choline PET/CT Determining a Change in Clinical Management of a Patient with Recurrent Prostate Adenocarcinoma

**DOI:** 10.1155/2014/297031

**Published:** 2014-08-13

**Authors:** Carmelo Tuscano, Elvio Grazioso Russi, Said Al Sayyad, Stefano Pergolizzi

**Affiliations:** ^1^Radiation Oncology Department, AO “Bianchi Melacrino Morelli”, Via Giuseppe Melacrino 21, 89124 Reggio Calabria, Italy; ^2^Radiation Oncology Department, University Teaching Hospital AO “S. Croce e Carle”, Via M. Coppino 26, 12100 Cuneo, Italy; ^3^Radiation Oncology Department, University of Messina, Via Consolare Valeria, 98100 Messina, Italy

## Abstract

A 66-year-old-man underwent a PET/CT scan after a biochemical relapse for a prostate cancer previously treated with a laparoscopic surgical procedure which revealed a focal uptake in the posterior wall of sigmoid colon. The biopsy demonstrated a colon cancer with mucinous differentiation producing a shift in clinical priority. To the best of our knowledge this is the first report in the English literature describing the detection by ^18^F-choline PET/CT of a colorectal cancer with mucinous differentiation.

## 1. Introduction

Whole body positron emission tomography/computed tomography (PET/CT) with [(11)C]- and [(18)F]-labeled choline derivatives has emerged as a promising molecular imaging modality for the evaluation of prostate cancer [1].

The rationale for using choline derivatives as oncologic PET tracers is based on the observation that choline transport and phosphorylation are upregulated in most cancers, including prostate cancer [2].

## 2. Case Report

We report a case of a 66-year-old man with biochemical recurrence of prostate cancer after a laparoscopic surgical procedure with PSA value of 5.2 ng/mL at the time of investigation by fluorine-18-choline (^18^F-choline) PET/CT.

**Figure 1:**
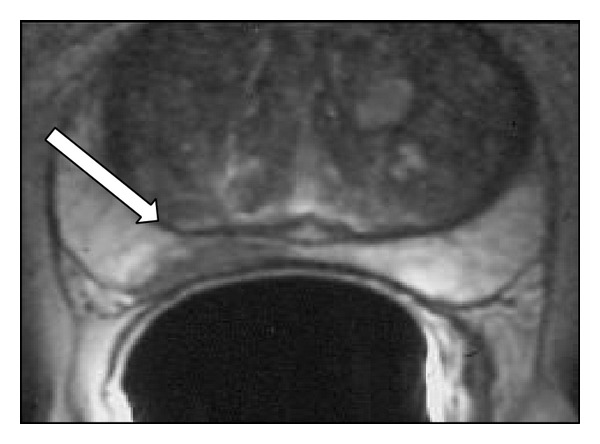
The presurgical high resolution T2w MRI imaging with a dark intraprostatic hypointense spot (white arrow) in the context of the right lobe relatively hyperintense peripheral zone, demonstrating an apparently organ-confined neoplastic disease.

At the time of the diagnosis, on December 2012, the total PSA value was 9.8 ng/mL and free/total PSA ratio was 0.13, indicating a concrete risk for the presence of a prostate carcinoma as demonstrated by Ito et al. in a paper frequently used in our center as a solid clinical reference [3]. At digital rectal examination (DRE) it was possible to appreciate a hard area at level of the right prostate lobe. Subsequently the patient underwent 12 cores transrectally guided by ultrasounds biopsy that demonstrated a prostate adenocarcinoma on the specimens obtained from right prostate base (Gleason score 3 + 3) and right mid prostate (Gleason score 3 + 4). At the time of the diagnosis (December 2012), at our Radiation Oncology Department, we used a simple magnetic resonance imaging (MRI) protocol to exclude the extracapsular extension of the neoplasia (clinical T3a) or the extension at the level of seminal vesicles (clinical T3b) four weeks after the biopsy to avoid the possible artifacts due to the presence of hematoma. The MRI study was performed using a 1.5 T system (GE Healthcare, Milwaukee, WI) with an endorectal coil (Medrad Inc., PA) and an external pelvic coil. Multiplanar T2-weighted images were obtained with FSE technique: TR/TE 5000/104 ms, 3 mm section thickness, 3.5 mm interleaved acquisition, 10–12 cm field of view, and 256 × 256 matrix. Axial T1-weighted (TR/TE 500/8 ms) images were also obtained. The MRI, on the T2w sequences, demonstrated with a high degree of overlapping with data obtained by the multicore biopsy a focal intraprostatic dark spot on the context of the relative hyperintense peripheral zone involving more than one-half of the right lobe [Figure 1] compatible with a clinical T2b prostate cancer. On January 2013 the patient underwent a laparoscopic surgical procedure consisting in resection of prostate gland and periprostatic fat at the level of obturator nodes. The pathologic specimen demonstrated the presence of microinfiltration of extracapsular tissue (pT3a) at the level of the more medial portion of the right prostate lobe. The highest value of GS was confirmed to be (3 + 4). The resection margins were not infiltrated. We were in front of the not unusual, in our experience, case scenario in which the definitive postsurgical pathological analysis demonstrates an understaging by the presurgical diagnostic procedures. However the PSA value measured two weeks after surgical intervention was <0.01 ng/mL and, after a discussion with the clinicians, the patient decided to postpone an eventual adjuvant radiotherapy for PSA values >0.4 ng/mL as suggested by Stephenson et al. [4]. Unfortunately the patient, despite the clinicians severe indications, from February 2013 to April 2014 performed just two measures of PSA values (on June 2013 with PSA = 0.2 ng/mL and on April 2014 with PSA = 5.2 ng/mL) demonstrating the presence of an aggressive cancer with very short PSA doubling times.

**Figure 2:**
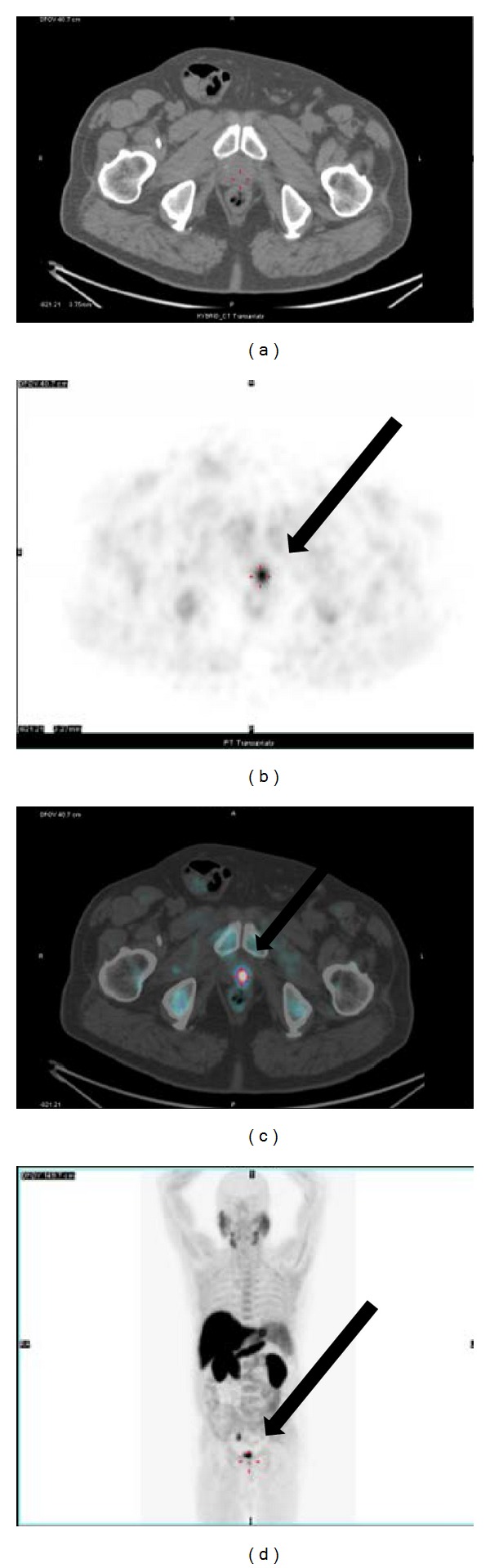
A 66-year-old man had restaging after biochemical relapse of a prostate cancer with ^18^F-choline PET/CT scan that revealed a focal uptake at level of the prostate surgical bed as shown on PET and fused PET/CT ((b), (c) black arrows).

**Figure 3:**
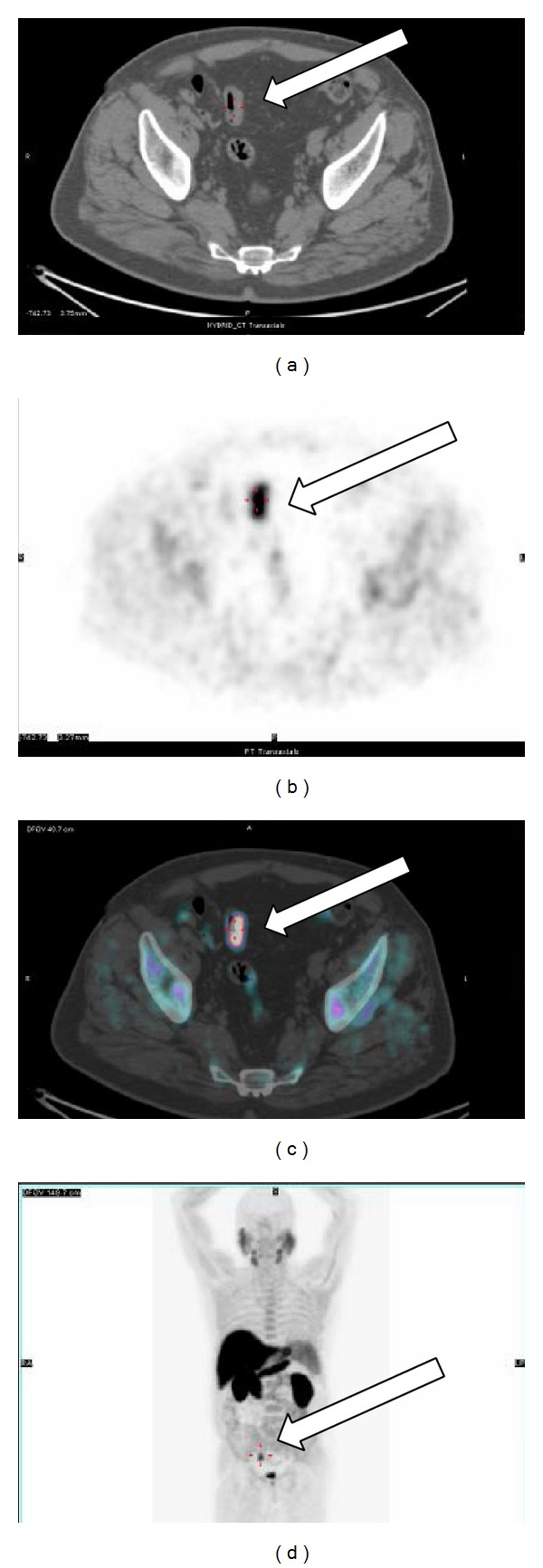
Incidental high SUV (SUVmax = 12) focal uptake at level of the thickened posteromedial wall of sigmoid colon was observed on CT, PET, and fused PET/CT ((b)–(d) white arrows). An endoscopic biopsy demonstrated a poorly differentiated mucinous colon cancer.

**Figure 4:**
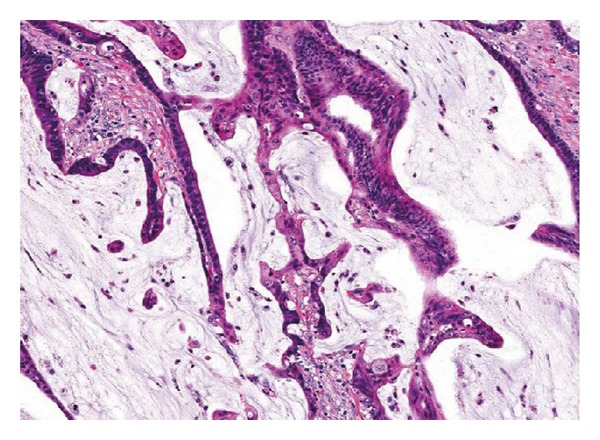
Mucinous adenocarcinoma showing abundant extracellular mucin (original magnification ×200), courtesy of the Department of Human Pathology of the Hospital “Bianchi Melacrino Morelli” in Reggio Calabria (Italy).

^18^F-choline PET/CT performed soon after the April 2014 PSA measurement demonstrated a focal uptake at level of prostate surgical bed (SUVmax = 9.4) suggestive for local recurrence [Figure 2]. The same scan revealed the presence of an intense uptake (SUVmax = 12) located at the posterior wall of sigmoid colon [Figure 3]. The endoscopic biopsy demonstrated the presence of a high grade (G3) mucinous colon carcinoma. The incidental finding shifted the clinical priority for this patient that was directed to surgical resection before treating the prostate cancer relapse. The mucinous colon cancer had the macroscopic aspect of a sessile polyp with a base of implant of about 3.5 cm into the intestinal wall. Microscopically [Figure 4] it was possible to appreciate the typical aspect with large glandular structures and with pools of extracellular mucin. This special type of colorectal carcinoma is defined by >50% of the tumor volume composed of extracellular mucin [5]. The tumor detected by the ^18^F-choline PET/CT invaded the muscularis propria (pT2) without involvement of the pericolorectal fat and of locoregional nodes (pN0). A postsurgical whole body contrast enhancement CT scan did not show the presence of lesion compatible with secondarisms by this colorectal cancer. Considering the usual aggressive behavior of mucinous colorectal cancer it is possible to affirm with a relative degree of confidence that ^18^F-choline PET/CT was able to detect this neoplasia at an early phase of its natural history.

## 3. Discussion

Radiolabeled choline PET/CT scan is a molecular imaging modality that has demonstrated its clinical usefulness in the management of recurrent prostate cancer [6, 7]. Pelosi et al. [8] demonstrated the diagnostic sensitivity in postprostatectomy patients to be 20, 44, and 81.8% at PSA levels of ≤1, >1 and ≤5, and >5 ng/mL, respectively. Under the radiation oncologist point of view choline PET/CT is a valuable tool that can be used for GTV definition in the RT planning process in the salvage RT setting [9].

The potential of radiolabelled choline in the detection of some benign lesions such as parathyroid adenoma and thymomas or other diseases such as malignant tumors and lymphomas [10]has been previously demonstrated.

Two previous reports [10, 11] have demonstrated the possibility of colon cancer detection in patients who underwent a scan with radioactive choline.

**Figure 5:**
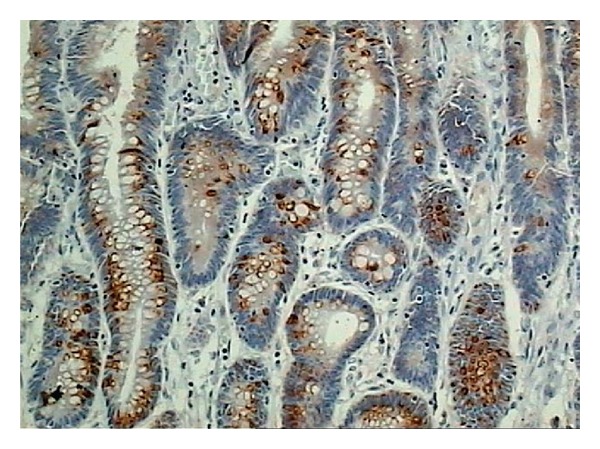
Immunohistochemical assay demonstrating the high density of the mucin 2 protein (MUC2) as typical biochemical feature of the mucinous colorectal cancer, courtesy of the Department of Human Pathology of the Hospital “Bianchi Melacrino Morelli” in Reggio Calabria (Italy).

Mucinous colorectal carcinoma (CRC) represents a subtype of colorectal carcinoma which is characterized by abundant amount of extracellular mucin. Mucinous CRC is associated with a higher expression of MUC2 [Figure 5] and MUC5AC but a lower expression of MUC1. The differential expression of mucins has been related to altered risk of metastasis and death. Recently, mucins have been used as targets for molecular therapy and as a source of immune therapy. Mucinous differentiation is associated with other specific genetic and molecular features such as increased BRAF mutation rate and microsatellite instability [12].

In our case, the incidental detection of a high SUV uptake at level of sigmoid colon in a whole body ^18^F-choline PET/CT followed by an endoscopic biopsy that demonstrated a poorly differentiated (G3) mucinous CRC produced a shift in clinical priority for the patient that was at first directed to surgical resection of the intestinal cancer.

To the best of our knowledge this is the first report in the English literature describing the detection by ^18^F-choline PET/CT of a colorectal cancer with mucinous differentiation.

It is reasonable to further investigate the correlation between colon focal uptake of ^18^F-choline and positive endoscopic biopsy for colorectal cancer.
